# Bone Grafts and Substitutes in Dentistry: A Review of Current Trends and Developments

**DOI:** 10.3390/molecules26103007

**Published:** 2021-05-18

**Authors:** Rusin Zhao, Ruijia Yang, Paul R. Cooper, Zohaib Khurshid, Amin Shavandi, Jithendra Ratnayake

**Affiliations:** 1 Department of Oral Science, Faculty of Dentistry, University of Otago, 310 Great King Street, Dunedin 9016, New Zealand; zharu032@student.otago.ac.nz (R.Z.); yanru988@student.otago.ac.nz (R.Y.); p.cooper@otago.ac.nz (P.R.C.); 2 Department of Prosthodontics and Dental Implantology, College of Dentistry, King Faisal University, Al-Ahsa 31982, Saudi Arabia; zsultan@kfu.edu.sa; 3 BioMatter Unit—École Polytechnique de Bruxelles, Université Libre de Bruxelles (ULB), Avenue F.D. Roosevelt, 50—CP 165/61, 1050 Brussels, Belgium; amin.shavandi@ulb.be

**Keywords:** replacing tooth loss, dental implant, bone defects, bone reconstruction, bone graft, bone tissue engineering, natural and synthetic bone substitutes

## Abstract

After tooth loss, bone resorption is irreversible, leaving the area without adequate bone volume for successful implant treatment. Bone grafting is the only solution to reverse dental bone loss and is a well-accepted procedure required in one in every four dental implants. Research and development in materials, design and fabrication technologies have expanded over the years to achieve successful and long-lasting dental implants for tooth substitution. This review will critically present the various dental bone graft and substitute materials that have been used to achieve a successful dental implant. The article also reviews the properties of dental bone grafts and various dental bone substitutes that have been studied or are currently available commercially. The various classifications of bone grafts and substitutes, including natural and synthetic materials, are critically presented, and available commercial products in each category are discussed. Different bone substitute materials, including metals, ceramics, polymers, or their combinations, and their chemical, physical, and biocompatibility properties are explored. Limitations of the available materials are presented, and areas which require further research and development are highlighted. Tissue engineering hybrid constructions with enhanced bone regeneration ability, such as cell-based or growth factor-based bone substitutes, are discussed as an emerging area of development.

## 1. Introduction

A bone graft is defined as a living tissue capable of promoting bone healing, transplanted into a bony defect, either alone or in combination with other materials [1,2]. A bone substitute is a natural or synthetic material, often containing only a mineralized bone matrix with no viable cells, that is able to achieve the same purpose [3]. Bone grafts and substitutes have been used in the medical field for centuries, with the first recorded use of bone grafts in 1682, where a cranial defect was successfully restored using a cranial bone graft from a deceased dog [4]. According to the US Food and Drug Administration (USFDA), bone grafts are classified as Class II devices (bone grafts filling the bony voids and defects) and Class III devices (bone graft containing drugs). The use of bone grafts and substitutes in dentistry have markedly increased in recent years due to advancements in dental implantology and the growing need for repair of craniofacial bony defects. These bony or skeletal defects may arise from trauma, periodontal disease, surgical excision, cranioplasty, infection or congenital malformations, and oral cancer [2]. The most common observation of insufficient quantity of bone in dentistry is following tooth loss, where rapid resorption of alveolar bone occurs due to an absence of intraosseous stimulation that would typically occur via the periodontal ligament fibers [5]. Successful placement of dental implants requires sufficient alveolar bone dimensions, that is, at least 10 mm in height and 3 mm to 4 mm in diameter [6]. It has been estimated that up to 50% of all dental implant procedures currently performed will involve the use of bone grafts [7]. Globally, current statistics indicate that approximately 2.2 million bone graft procedures, costing an estimated US$664 million by 2021, are being performed each year, with the number of operative procedures for repairing bony defects estimated to grow by approximately 13% annually [8]. As of 2018, the market value for dental bone substitutes has been estimated to be worth approximately US$493 million and is projected to grow to approximately US$931 million by 2025, at a combined annual growth rate of 9.5% [9]. Despite this widespread use of bone graft and substitute materials globally, there are still limitations that remain associated with currently used materials. These primarily involve the use of allografts, the transfer of grafting materials between two genetically unrelated subjects; and autografts, the transfer of grafting material from one body site to another within the same subject [2]. None of the products in the market currently possesses all the ideal properties for a bone substitute material including low patient morbidity, ease of handling, low immunogenicity, low cost and angiogenic potential [10,11,12]. The disadvantages of autografts include the lack of availability of graft tissue, associated pain, morbidity at the donor site and the need for two operative procedures; whereas disadvantages of allografts include rejection of the donor tissue by the recipient’s immune system and concerns with transmission of diseases, such as HIV and hepatitis [8,13]. In recent years, there has been an increased drive in the market to use newer bone grafting materials, such as bone substitute products, despite little evidence-based research for indications and safety [10]. Thus, these matters of concern, along with continued marked increases in demand for bone graft materials and the global ageing population strongly indicate a need for further research into the development of novel materials used for bone grafting procedures [8,9,12].

This literature review reports on the dental bone graft and substitute materials that are currently available commercially, their limitations, and the potential development of promising alternatives brought about by the emergence of synthetic bone substitutes in recent decades. We aim to highlight the gap between what is currently available in the market and what would be considered an ideal bone substitute material of choice in the future and identify promising areas of further research to allow for the development of novel bone substitute materials with more desirable biological and mechanical properties. Therefore, this review will provide an update on current bone graft and substitute materials used in dentistry, their relative efficacies and shortcomings and future directions for study.

## 2. Characteristics of an Ideal Bone Grafting Material

**Figure 1:**
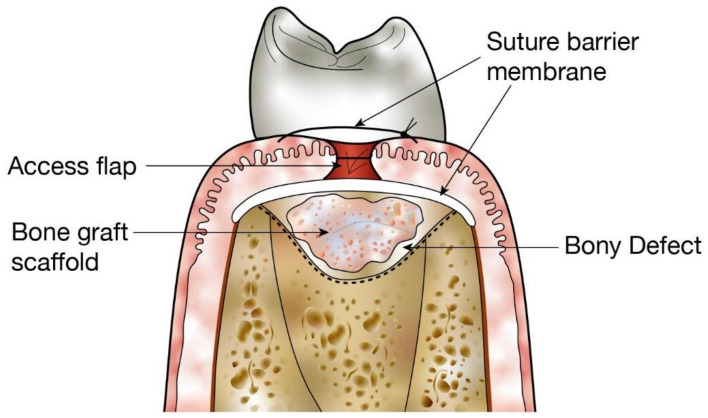
Use of structural scaffolds to restore bony defects. Diagram shows placement of a bone graft scaffold within a bony defect in alveolar bone following surgical generation of an access flap.

The main function of bone grafts is to provide mechanical support and stimulate osteo-regeneration, with the ultimate goal of bone replacement [10]. The four fundamental biological properties of osseointegration, osteogenesis, osteoconduction, and osteoinduction, are paramount in performing this role effectively [11,14]. The ability of a grafting material to chemically bond to the surface of the bone in the absence of an intervening fibrous tissue layer is referred to as osseointegration. Osteogenesis refers to the formation of new bone via osteoblasts or progenitor cells present within the grafting material, and osteoconduction refers to the ability of a bone grafting material to generate a bioactive scaffold on which host cells can grow [1,15]. This structure enables vessels, osteoblasts and host progenitor cells to migrate into the interconnected osteomatrix (Figure 1). Osteoinduction is the recruitment of host stem cells into the grafting site, where local proteins and other factors induce the differentiation of stem cells into osteoblasts [16]. Multiple growth factors influence this process, including platelet-derived growth factors (PDGFs), fibroblast growth factors (FGFs) and transforming growth factors-β (TGFs-β). These four fundamental properties enable new bone formation which occurs in parallel to direct osseous interconnection [17,18].

Additionally, various other properties will influence the success rate of a bone graft. These include, but are not limited to, biocompatibility, bioresorbability, sterility, structural integrity, adequate porosity for vascular ingrowth, plasticity, ease of handling, cost, and compressive strength [17]. A combination of these factors forms the basis for their use, adequate long-term tolerance by host tissues, and increased chances of successful osteo-regenerative processes occurring [17]. 

Studies have found that almost all current bone graft and substitute materials primarily serve as a structural framework for osteo-regenerative processes to occur, thus they only satisfy the osteoconductivity component of the ideal characteristics discussed previously [17,19,20]. Additionally, potential issues persist relating to graft vs. host responses for all current non-autograft-derived materials. This forms a significant area for improvement in the subsequent development of novel bone substitute materials in the future.

## 3. Classification of Dental Bone Graft and Substitute Materials

**Figure 2:**
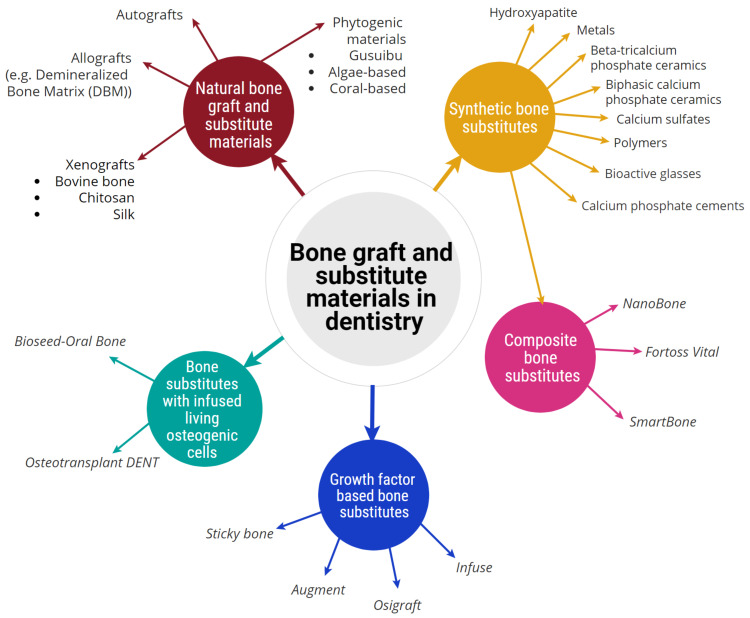
Classification of bone graft and substitute materials used in dentistry, broadly classified into five categories and showing their associated sub-categories.

There are two primary methods of classifying bone graft and substitute materials, based on the tissue source or the material group [5,17]. Bone graft and substitute materials currently used in the dental field have been broadly classified into five categories (Figure 2). This section discusses various dental bone graft and substitute materials, which are currently used to fill bony voids, and augment or reconstruct periodontal and alveolar bone defects.

### 3.1. Natural Bone Graft and Substitute Materials

Materials of natural origin are defined as those that have been derived from a living source without modification. These materials can be further subdivided into four subcategories: autografts, allografts (such as demineralized bone matrix (DBM)), xenografts, and phytogenic materials [11,17,18]. It has been estimated that up to 90% of all bone grafting procedures performed worldwide use a natural bone graft or substitute material [17]. Characteristics of commercially available natural bone graft or substitute materials used in the dental field are displayed in Table 1.

#### 3.1.1. Autografts 

Autografts are commonly obtained from intraoral and extraoral sites from the same individual, such as the mandibular symphysis, mandibular ramus, external oblique ridge, iliac crest, proximal ulna, or distal radius, due to being good sources of cortical and cancellous bone [2]. Autograft bone harvested from the mandibular ramus is associated with more minor downstream complications compared with other intraoral sites although this presents a risk of damage to the inferior alveolar nerve. Mandibular ramus grafts are appropriate for use when the sites requiring augmentation are less than 4 mm in thickness and span a maximum of four teeth [31,32]. There are no histocompatibility and immunogenicity issues associated with autografts, therefore they represent the highest degree of biological safety. However, there are several downsides associated with autografts, such as the requirement for a secondary surgical visit, donor site injury and the potential for scarring. Additionally, autografts have been associated with higher surgical costs, more significant surgical risks, e.g., excessive bleeding, infection, inflammation and pain, limiting their application to relatively smaller bone defects. Thus, in large craniofacial defects, autografts may not represent a viable option [10,12]. 

Cancellous bone is most commonly used for autografts and contains osteoblasts and progenitor cells with considerable osteogenic potential. They possess relatively large trabecular surfaces, which facilitate establishment of an osteoinductive environment by encouraging revascularization and incorporation into the recipient site. In contrast, cortical bone lacks osteoblasts and osteogenic cells; instead, it provides structural-mechanical integrity and promotes bone healing through osteoconduction. Cortical grafts are slower to integrate relative to cancellous grafts due to their limited revascularization potential. Therefore, to maximize bone remodeling performance and healing potential, a combination of cancellous and cortical bone is used [18]. Despite the development of numerous bone substitutes over recent years, autografts remain the gold standard for grafting materials as they are still the only graft material that possesses all of the four fundamental biological properties required [33]. In dental applications, even though other bone substitutes are routinely used for management of localized alveolar bony defects and maxillary sinus bone grafting, autografts in block forms are still routinely used in alveolar ridge augmentation procedures. As very few bone substitutes can produce a volume of newly formed bone comparable to that produced by autograft materials therefore, autografts remain the material of choice for more complex augmentation procedures, such as posterior mandibular edentulous reconstruction [33,34,35]. This is because autogenous block grafts can increase the bone quality and quantity in a predictable manner, allowing for placement of implants with maximal diameters which facilitate strength distribution for long-term survival [33,34].

#### 3.1.2. Allografts

The primary alternative to an autograft is the use of allograft materials, which can be obtained from either a compatible living donor or from cadaveric bone sources [18]. Allograft materials can be prepared in three primary forms—fresh, frozen, or freeze-dried. Fresh and frozen allograft materials possess superior osteoinductive properties but are rarely used nowadays due to the higher risk of a host immunogenic response, limited shelf life, and increased risk of disease transmission (Table 1). Further processing of allograft material through freeze-drying can increase the shelf life of the material and decrease the immunogenicity, though at the cost of decreased osteoinductive potential, decreased structural strength, and osseointegration [18]. 

**Figure 3:**
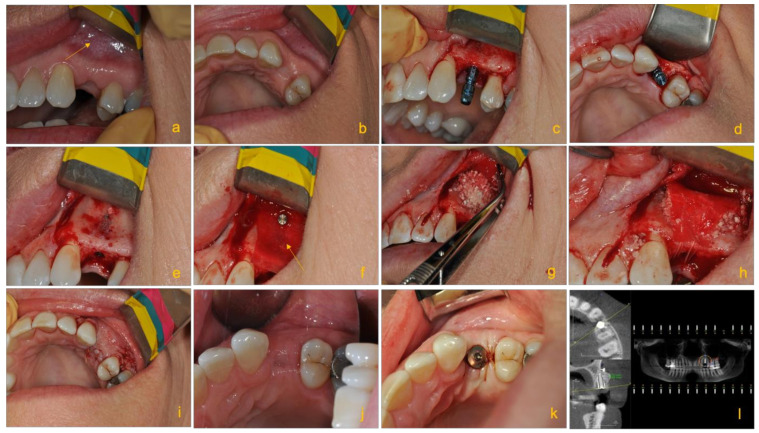
Clinical images illustrating the pre- and post-operative (post-op) procedures of an edentulous patient treated with an implant with guided bone tissue regeneration. Images from left to right show: (**a**) the edentulous site after the tooth was extracted; bone defect in the form of buccal concavity is visible in the apical aspect of 24 (yellow arrow). (**b**) Occlusal shot of the edentulous site showing the buccal concavity. (**c**) The correct positioning of the implant (Straumann Bone Level Tapered 3.3 × 10 mm implant). (**d**) Proper bucco-palatal positioning of the implant. (e) Decorticated area to prior to placement of the bone and complete absence of the buccal bone at the apical part of the implant. (**f**) The placement of Straumann Flex Membrane fixed and stabilized by tacks (AutoTac by BioHorizons Canada). (**g**) Applying bone graft particles comprising of a mixture of Allograft and Xenograft (both from Straumann^®^) packed at the buccal bone defect. (**h**) Periosteal sutures used to stabilize and fix the bone graft inside the membrane, which ensures immobilization of graft resulting in optimal bone regeneration vs. fibrous tissue formation. (**i**) Primary closure of the site. (**j**) Showing post-op. Primary closure is intact. (**k**) The implant after second stage and osseointegration check. (**l**) Five months later, post-op cone beam computed tomography (CBCT) illustrating the final bone healing prior to second stage and osseointegration check of the implant. The post-op CBCT revealing a gain of over 5 mm of bone (courtesy of Dr. Mohammad A. Javaid, Periodontist, British Columbia, Canada).

In recent decades, the use of allograft materials has often been preferred [36]. This is largely due to the alleviation of many of the major concerns associated with autografting procedures described previously, especially in large bony defects. However, limitations persist relating to the risk of infectious disease transmission, such as for human immunodeficiency virus (HIV) and Hepatitis B and C. Studies have found that there is ~8% prevalence of unknown diseases in osteoarthritic femoral heads removed during hip arthroplasty [37]. These concerns can generally be alleviated through tissue processing such as sterilization, mechanical debridement, ultrasonic washing and gamma irradiation [15]. Allografts have been successfully used in combination with xenografts for guided bone regeneration (GBR) in bone augmentation procedures (Figure 3). 

Allografts exhibit good histocompatibility and are found available in various forms, from whole bone segments, cortico-cancellous, and cortical pieces to chips, wedges, pegs, powder, and DBM (Table 1). Allograft materials can also be produced in custom shapes to satisfy the requirements of the recipient sites. Cancellous autografts and allografts however have poor mechanical strength and cancellous allografts also exhibit inadequate healing capacity, as the tissue processing techniques, including treatment with alcohol, acetic acid, or nitric acid, reduce the materials’ osteoinductive capabilities [18]. Cancellous allografts can cause local host inflammatory response, resulting in fibrous tissue formation that can interfere with the new bone formation. Cortical allografts are similar to cortical autografts and possess considerable mechanical strength, and serve largely to provide a mechanical scaffold for bone healing processes to occur following an initial inflammatory cascade [11,18]. In dental applications, allografts have been used to fill periodontal, maxillary, and mandibular defects (Table 1). Block allografts have routinely been used to restore deficiencies in alveolar ridge height or severe ridge atrophy to allow for sufficient bone height for implant placement [38,39,40]. In recent years, concerns relating to the shortage of tissue supply, as well as findings of high failure rates following long-term use have led to a decline in the application of allograft materials. Moreover, increased regulatory restrictions regarding the use of allograft materials in Europe have driven a shift towards the use of more synthetic grafting materials over allograft materials [3,41].

Demineralized bone matrix is an allograft derivative, which is acid-treated to remove the mineral mesh [11,15,18]. This demineralization process exposes the underlying inner bone matrix, rich in bone morphogenic protein (BMP) and growth factors, such as TGF-β and FGF. These growth factors can stimulate the differentiation of mesenchymal stem cells (MSCs) into osteoblasts, conferring an osteoinductive capacity greater than that of cancellous or cortical allografts [42]. Despite the remaining osteoinductive molecules, the osteoinductive potential of DBM is highly dependent upon tissue preparation techniques, such as alcohol, lactic acid, acetic acid, and nitric acid treatment—all of which adversely affect its osteoinductivity [10,18]. Following demineralization processing, the original tissue’s trabecular framework remains, which allows for bone formation following vascular ingrowth and infiltration of progenitor cells [10]. Freeze-dried DBM was one of the earliest commercially available preparations of DBM and has been used in a variety of forms, such as blocks, particulates and powders, providing an osteoconductive matrix (Table 1) [42]. In recent years, DBM has been increasingly used in dental applications with added excipients that act as transport vehicles, such as glycerol, starch, hyaluronic acid, collagen, and saline. This preparation allows for improved handling and adaptability due to the hardening of the mixture and its components. An additional benefit of Freeze-dried DBM is the slow release of BMPs which have been shown to possess the ability to induce bone regeneration, increasing the osteoinductive potential [15,43]. Demineralized bone matrix is relatively easy to handle with minimal immunological rejection due to the elimination of the antigenic surface structure of the bone during acid-treatment; they also exhibit the conventional benefits of allograft materials [10,11,15,18]. However, DBM provides a lack of structural support and thus possesses poor mechanical properties. Therefore, the use of DBM is only limited to filling bone defects and is generally used in combination with other allografts, BMPs or composite bone substitute materials [2,3,10]. Additionally, the risk of infection and immunologic reaction associated with allograft-based materials encouraged researchers to explore new materials that stimulate bone healing from other sources such as plant-based or synthetic materials (Table 1) [44]. Human DBM in putty form has been successfully used to preserve and restore alveolar bone height and thickness following tooth extraction, resulting in the formation of mineralized and mature bone six months after grafting [23,45]. 

Collagen-based materials, such as extracellular bone matrix are another allograft-derived material available in the market. These materials can provide a favorable environment for new bone formation through mineral deposition, vascularization, and growth factor adhesion. However, collagen-based materials present a high potential for adverse immune reactions and possess poor structural integrity. Therefore, it is generally considered a poor graft substitute alone, although it can improve osseointegration when incorporated with BMPs or a hydroxyapatite carrier [15,46,47].

#### 3.1.3. Xenografts

As discussed previously, autografts and allografts possess inherent limitations despite their excellent success rates in bone grafting practice. Therefore, natural bone substitutes have been developed to encourage improved osteogenic, osteoconductive, and osteoinductive potentials by creating a favorable bone growth microenvironment [48]. Xenografts are grafting materials that are derived from a genetically unrelated species from the host [15]. The most common source of xenograft materials in the dental field is deproteinized bovine bone which is commercially available as *BioOss*^TM^ (Table 1). Bovine bone is treated with a stepwise annealing process followed by chemical treatment with NaOH to produce a porous hydroxyapatite (HA) material containing only the inorganic components of bovine bone. The resulting porous structure highly resembles that of human bone and can provide good mechanical support and stimulate bone healing through osteoconduction. The porous structure exhibits a vast surface area, and promotes the growth of new blood vessels via angiogenesis which enhances bone growth. Bovine bone substitutes have been used extensively in maxillary sinus lifting and implant procedures due to their superior stability and low immunogenicity (Table 1) [15,49]. Studies have found that maxillary sinus defect sites grafted with *BioOss*^TM^ resulted in 39% new bone formation after 6 months, which was comparable to 40% new bone formation following grafting with autograft bone after the same time period. Furthermore, they found that 31% of grafted *BioOss*^TM^ remained at the graft site, compared with just 18% of the autograft bone [15,50]. These statistics suggest that the efficacy of *BioOss^TM^* in stimulating new bone formation closely matches, if not exceeds, that of autograft bone. A 5-year prospective follow-up study conducted by Ozkan et al. (2011) also found that sufficient quality and volume of bone, allowing for predictable simultaneous placement of implants, resulted from bovine bone grafts following one-stage maxillary sinus augmentation procedures [51]. Clinically, BioOss has proven to be a valuable bone substitute material providing good quality of new bone and promising long-term survival rates following dental surgery [52].

Other commercially available products based on bovine bone are also available, such as *OsteoGraf^TM^* and *Cerabone^TM^* (Table 1). Both of these products are high temperature treated, thus eliminating all organic components, which result in a product that possesses low immunogenicity. Like *BioOss^TM^*, these products exhibit very similar structural and biochemical properties to human bone and can act as effective osteoconductive grafting materials [17]. 

A promising xenograft material currently being researched is chitosan, a naturally occurring polymer derived from the exoskeletons of crustaceans composed of glucosamine and N-acetylglucosamine [53]. Chitosan is able to stimulate bone regeneration by providing a structural scaffold that supports osteoblastic activity, the formation of mineralized bone matrix and inducing differentiation of MSCs into osteoblasts in various in vitro environments [48]. Chitosan is available in a variety of forms, including beads, films, hydrogels, and more complex structures, such as porous scaffolds (Table 1). Due to the poor mechanical properties exhibited by chitosan, it is often combined with other materials such as gelatin, calcium phosphates and bioglass to provide more desirable properties [54,55,56,57]. Wattanutchariya and Changkowkai found that mixing chitosan with gelatin and hydroxyapatite (HA) produces a porous scaffold with more desirable properties, including decreased degradability and an open pore structure conducive to cell attachment and vascularization [58]. Chitosan-based bone substitute materials also possess other beneficial properties, such as low immunogenicity, fibrous encapsulation, structural versatility and a hydrophilic surface that promotes cell adhesion and proliferation (Table 1) [48]. The versatility associated with chitosan-based bone substitute materials is what indicates it as such a promising alternative to the conventional gold-standard of autografts. Recent studies in the dental field have reported the successful use of chitosan-based materials as a membrane for GBR, guided tissue regeneration, coating implant surfaces, periodontal regeneration and restoring alveolar bone height (Table 1) [59,60].

Silk is a natural biopolymer obtained from the silkworm *Bombyx mori.* It is predominantly composed of the proteins, fibroin and sericin. After removal of sericin through degumming, silk fibroin (SF) is commonly used as a bone scaffold in sponge, fibers, film and hydrogel forms (Table 1) [61,62,63]. SF demonstrates excellent biocompatibility, degradability, tissue integration, oxygen and water permeability (Table 1). Recent research has shown that silk’s favorable biological properties enable its use as a membrane for GBR despite its poor mechanical properties. In several clinical trials performed in 2016, patients who received a silk mat membrane following extraction of impacted mandibular third molars displayed a significant gain of new bone of approximately 4mm, six months following the grafting procedures [61,64,65]. SF has been indicated for use as a GBR membrane following tooth extraction, cyst/tumor excision, and deficient alveolar bone for implant placement [66]. SF-based membranes have been shown to exhibit excellent tensile strength, good osteogenic potential and good mechanical properties in several in vivo studies (Table 1) [65,67]. HA can also be blended with an integrated membrane of SF and chitosan to improve the membrane’s mechanical strength and stability [61]. Despite the promising outlook for the many xenograft materials described, there still remains some limitations associated with use xenograft bone substitutes. These include the variable resorption rates, lack of viable cells and biological components and the need for tissue treatment processes which enable the retention of osteoinductive cells [12].

#### 3.1.4. Phytogenic Material

Phytogenic materials are bone substitute materials obtained from a plant-based origin, such as Gusuibu, coral-based bone substitutes, and marine algae. Gusuibu is a traditional Chinese herbal medicine that has been used widely for the treatment of bone fracture and osteoarthritis in Chinese patients [11,15,68]. Gusuibu is the name of the dried rhizome of perennial pteridophyte *Drynaria fortunei.* This material has been shown to possess osteoinductive properties, increased alkaline phosphatase activity, and thus promotes bone calcification and remodeling processes (Table 1) [44]. Wong and Rabie found that when Gusuibu was integrated with a collagen carrier acting as a structural scaffold, new bone formation was increased by 24% all across the bony defect when compared with the grafted Guisuibu alone; and by 90% when compared with an absorbable collagen sponge routinely used as a carrier for growth factors (GFs) such as BMPs to induce bone regeneration [44]. These results show that the efficacy of Gusuibu in promoting new bone formation was comparable to that of the autograft material, suggesting that Gusuibu may present a promising viable bone substitute material when used with a collagen carrier. In dental applications, Gusuibu has been shown to accelerate bone remodeling following orthodontic tooth movement through promotion of osteoblastic activity, and cell culture studies have revealed that Gusuibu is able to mediate bone remodeling through regulation of osteoclast and osteoblast activity [69,70].

Coral-based bone substitutes are predominantly composed of calcium carbonate used either in its naturally occurring form or processed by heat treatment with ammonium phosphate and converted into crystalline HA which subsequently has minimal residual carbonate [3,10,71,72]. HA is a natural polymer of calcium phosphate obtained from bone or natural materials, such as coral, and widely used to promote bone healing due to its ability to act as a structural scaffold. The main issue associated with naturally occurring coralline HA is its brittleness and high resorbability; hence coral-based materials are most commonly used as crystalline HA in granule or block form to provide a structural framework very similar to that of trabecular bone (Table 1) [10]. Studies have shown that coralline HA improves vascularization compared with non-coralline HA and performs better than freeze-dried allograft materials in terms of promoting cell attachment [73,74]. In clinical practice, coralline HA is often integrated with autograft materials, or acts as a carrier for osteoinductive GFs such as BMPs to enhance bone healing in bony defects [10]. Recent experimental studies have investigated improving the biomechanical properties of coralline HA using a variety of mechanisms, such as doping with fluorine and zirconia, which aims to improve mechanical strength and the incorporation of strontium ions allowing for enhanced stimulation of bone formation and inhibition of bone resorption. Additionally, vascular endothelial growth factor (VEGF) coated coralline HA has recently been used in alveolar defect animal models, leading to enhanced vascularization and mineralization [75,76,77,78]. Coralline HA-based materials used in dentistry have varying pore sizes and exhibit good compressive strengths, low immunogenicity, good bone bonding capacity, however they have relatively low tensile strengths, brittleness and poor resorption (Table 1) [3,79]. These materials have been used for procedures such as maxillary sinus lifting, periodontal osseous defects and alveolar reconstruction for placement of dental implants (Table 1) [29,80,81].

*AlgiPore*^TM^ is a naturally occurring HA derived from marine algae that has been clinically used as a bone substitute material since 1988 [82]. This material possesses desirable properties such as good resorbability over time, a large surface area for protein adhesion, and low immunogenicity (Table 1). *AlgiPore*^TM^ is also able to act as a carrier for GFs and MSCs [83,84,85]. Despite extensive evidence in in-vitro, in-vivo and clinical studies of bone healing following *AlgiPore*^TM^ grafting, few studies have investigated its use in humans and even fewer have investigating modifications [27,83,86,87,88,89]. Recent developments have used *AlgiPore*^TM^ in combination with β-TCP, which claims to decrease resorption times whilst maintaining the volume support required for bone healing [82,90]. A 14-year longitudinal study conducted by Ewers found 95% implant survival rate in atrophic maxillae following sinus grafting procedures with *AlgiPore*^TM^ [27]. *AlgiPore^TM^* is considered to be a very favorable bone substitute material in relation to its bone filling capabilities due to its excellent biocompatibility, such as low immunogenicity, biodegradability and bone-bonding capacity (Table 1) [91]. Clinically, *Algipore*^TM^ has mostly been used as a space filler, in combination with other materials following tooth extraction to prevent ridge deformities (Table 1) [92]. 

### 3.2. Synthetic Bone Substitute Materials

To overcome potential immunogenicity and morbidity at donor sites, artificial synthetic bone substitute materials are generated to closely mimic the biological properties of natural bone. Despite this, currently available synthetic materials display only osteointegrative and osteoconductive properties [14]. Materials that fall into this category include calcium phosphate ceramics, such as hydroxyapatite (HA), tricalcium phosphate (TCP) and bioglass; metals, such as nickel-titanium; polymers, such as polymethylmethacrylate (PMMA), and polyglycolides and calcium phosphate cements [5,17]. Characteristics of commercially available synthetic bone substitute materials used in the dental field are described in Table 2.

#### 3.2.1. Hydroxyapatite (HA)

The chemical composition of HA closely resembles that of the inorganic component of bone, which enables it to be used as a bone grafting material [116]. However, synthetic HA do not contain trace amounts of Na^+^, Mg^2+^, K^+^ and Sr^+^, which are found in naturally derived HA, such as bovine bone, which influences various biomechanical reactions. Synthetic HA does not possess a microporous structure, as seen in bovine-derived HA [8]. Synthetic HA has a delayed resorption rate due to its relatively high Ca/P ratio and crystallinity. Another major concern associated with HA is its relatively low mechanical strength preventing it from being used at high load-bearing sites (Table 2). Previous studies have found that the quality and quantity of new bone formed following grafting with synthetic HA alone or in combination with a polymer was insufficient for preservation of alveolar ridge heights for placement of endosseous implants, maxillary sinus lifting and management of periodontal osseous defects [117]. Hence, the application of HA in dentistry is generally limited to the coating on implants, external fixator pins or in sites with low loading stress (Table 2) [11,116].

Recent advances in HA-based bone substitute materials have looked into producing nano-sized HA, which displays enhancesbiomechanical properties that more closely mimics the composition of natural bone. The rationale for development of these nano-sized materials include a much closer resemblance to bone extracellular matrix; a faster response to external environmental stimuli; enhanced delivery and controlled release of bioactive molecules, such as growth factors, allowing for enhanced osteo-regenerative properties [118,119]. Nanocrystalline HA exhibits improved biological performance and dissolution compared with its conventional forms of HA [120]. The nanostructure allows for a larger surface to volume ratio, promoting more effective adhesion, proliferation, and differentiation of osteogenic progenitor cells; improves sinterability and enhances densification resulting in improved fracture toughness and other mechanical properties [116,121,122]. Despite significant improvements in performance across all domains compared with conventional forms of HA, there remains insufficient evidence to support nanocrystalline HA’s widespread use [116,118].

#### 3.2.2. Tricalcium Phosphate Ceramics (β-TCP) 

TCP possesses two crystallographic forms, α-TCP and β-TCP [68,123]. β-TCP is a type of calcium phosphate material widely used as a bone substitute material over many years. It exhibits faster biodegradation and absorption compared with HA due to its lower Ca/P ratio [11]. Pure phasic β-TCP possesses many desirable properties, such as its ease of handling, radiopacity allowing monitoring of healing, good osteoconductivity due to macroporosity promoting fibrovascular ingrowth and osteogenic cell adhesion, good resorbability compared with bovine bone grafts and low immunogenicity and risk of disease transmission (Table 2) [15,124]. Whilst the interconnected porous structure of β-TCP allows for improved vascularization, it also results in the material’s poor mechanical strength under compression [10,11,15]. This results in β-TCP being unsuitable as a bone substitute however it is suitable for use as a filler in bony defects and repair at morphological sites [125]. It is commonly used to repair marginal periodontal and periapical defects and as a partially resorbable filler in alveolar bony defects (Table 2) [126]. Nakajima et al. found that the bone regenerative potential of β-TCP is comparable to that of freeze-dried bone allograft, deproteinized freeze-dried bone allograft and autograft materials [127]. However, the mechanical properties of this material limit its wider application [3].

#### 3.2.3. Biphasic Calcium Phosphate Ceramics (HA and β-TCP Ceramics)

Advancements in the previous few decades have attempted to develop a material that was able to harness both the resorbability of β-TCP as well as the osteoconductive potential of HA [128]. This resulted in the development of biphasic calcium phosphate ceramics, where β-TCP and HA are commonly used in association. Hence, the resulting more rapid and higher bone regeneration rates seen compared with the use of HA alone, and the greater mechanical properties than β-TCP alone, present the major benefits of using biphasic CP ceramics [3,129,130,131]. Additionally, the resorption and osteoconductivity of biphasic calcium phosphate ceramics is able to be controlled by altering the ratio of HA/β-TCP [128]. Despite the improvements in mechanical strength compared with β-TCP alone, biphasic CP ceramics still possess compressive strengths lower than that of cortical bone [3,132]. The use of biphasic CP ceramics has been indicated as a bone substitute in periapical surgery and has shown predictable clinical outcomes and complete alveolar bone healing over a two year period (Table 2) [125]. Hence, this material has shown the potential for bone healing via osteoconduction and osteoinduction processes which could be further explored in clinical practice. 

#### 3.2.4. Bioactive Glass 

Bioactive glasses (BAG) are a group of synthetic silicate-based ceramics composed of silicates coupled to other minerals, such as Ca, Na_2_O, H and P [3,11]. The original composition of bioglass consisted of silicon dioxide (SiO_2_), sodium oxide (Na_2_O), calcium oxide (CaO) and phosphorus pentoxide (P_2_O_5_), though this has been recently modified to a more stable composition through the addition of potassium oxide (K_2_O), magnesium oxide (MgO) and boric oxide (B_2_O) [11]. Upon exposure to body fluids during implantation, silicon ions can leach out and accumulate, forming a layer of HA on the surface of the material, which allows for adherence of osteogenic progenitor cells. The desirable properties of bioglass include good biocompatibility, osteoconductivity, antimicrobial activity and a porous structure promoting vascularization [3,11,133]. Recent research has shown that incorporation of various ions with BAG is able to enhance the material’s properties. Esfahanizadeh et al. found that zinc-doped BAG resulted in reduced biofilm formation for microbes associated with periodontal disease [134]. Additionally, another research group, Lovelace et al. found that silver-doped BAG showed controlled release of ions from the material, enhancing its antibacterial properties against *Porphyromonas gingivalis (P.g), Prevotella intermedia (P.i)* and *Aggregatibacter Actinomycetemcomitas (A. a).* These microbes play a central role in the destruction of periodontal tissues, leading to gum disease and peri-implantitis [134]. PerioGlas^®^ and UniGraft^®^ are examples of commercially available BAG products in the market, containing 90–710 µm particle sizes. The advantages of these products include relatively easy handling and adaptability to the defect site, and they have been successfully used in periodontal surgery to stimulate bone regeneration [135]. Further studies observed significant reductions in pocket depth and a regain in clinical attachment in thirteen patients treated with UniGraft^®^ bioactive glass for infrabony defects [136].

However, BAG can be brittle and possess low mechanical strengths and poor fracture resistance. Thus, their use in dentistry is limited to low-stress environments or in combination with other grafting materials (Table 2) [133,137]. Bioglass materials have been successfully used to augment the unilateral cleft alveolar bone, manage periodontal osseous defects and preserve alveolar bone following tooth extractions in orthodontic patients (Table 2) [138,139,140]. 

#### 3.2.5. Calcium Phosphate Cements (CPCs)

Calcium Phosphate Cements are generally two or three-component systems consisting of an aqueous component and a powder component commonly containing sintered CP material, such as α-TCP and HA. Mixing of the components results in a workable paste which hardens in situ in a self-setting manner to form HA nanocrystals at room temperature [17,141]. The main advantages of CPCs include their self-setting ability, the ability to shape the paste into the defect site, their ability to replicate the structure and composition of bone in a repeatable manner, their high biocompatibility, availability in different forms for different types of bony defects and their osteoconductive properties [3,11,141]. However, CPC lacks a macroporous structure which limits the speed of cell adhesion, fluid exchange and restorability (Table 2) [15,141]. Additionally, the potential of an incomplete setting reaction resulting in an inflammatory reaction presents a major drawback associated with CPCs. Recent research has aimed to address these shortcomings and strategies used. These include the development of pre-fabricated 3D-printed CPC scaffolds and improved CPC injectability through various mechanisms including the addition of viscous binders such as chitosan, gelatin and hyaluronic acid; optimizing the particle size, distribution, shape, and inter-particle interactions of the CPC powder; regulation of the setting reaction and modification of external factors like syringe and needle sizes [142,143,144]. Furthermore, research has focused on improving the material properties of CPC products by doping CPCs with various ions, such as silicon and strontium to improve osteoconductivity; incorporating bioactive glass to improve bioactivity and infusion with growth factors and stem cells to improve osteo-inductivity [145]. Lyu et al. demonstrated that using CPCs in addition to a collagen membrane delayed new alveolar bone formation at extraction sites [146]. In addition, CPCs are generally brittle when subjected to tensile and shear forces, and thus they are indicated for use only in non-load bearing sites [3,18]. Another major concern associated with CPCs is the potential extrusion of the material into surrounding tissues causing potential damage to adjacent tissues [10]. In clinical dentistry, CPCs have been used as a filler for bony defects, reconstruction of bony fractures and dental implantology (Table 2). 

#### 3.2.6. Calcium Sulfates

Calcium sulfates refer to heated gypsum in powder form, eventually forming a crystalline structure known as alphahemihydrate [10]. When rehydrated, this powdered hemihydrate can form a workable paste that hardens in a self-setting manner allowing the material to be molded into bony defects of varying shapes and sizes [147]. Calcium sulfate has been extensively used as an osteoconductive scaffold for bone regeneration in the past [104,113,148]. Recent studies have shown that calcium sulfate also possesses osteoinductive properties due to the release of osteoinductive molecules stimulating bone healing [149,150]. This material’s primary advantages include low cost, high availability, high biocompatibility, short setting time and osteoconductivity. However, a major drawback associated with this material is rapid resorption times, which exceeds the rate of new bone formation, resulting in significant loss of mechanical properties at the defect site [3,150,151]. Additionally, the use of calcium sulfates has been associated with an increased risk of inflammation and infection; and thus, calcium sulfates are often mixed with other products such as antibiotics [3,10]. In dental applications, traditionally saliva and bleeding have presented a significant obstacle to the routine use of calcium sulfates. However, research in the previous decade sought to overcome this difficulty, resulting in the development of a biphasic form of the material containing approximately 33% hydroxyapatite, which allowed for the calcium sulfate to harden even in the presence of bodily fluids [152]. These advancements have enabled the application of use of calcium sulfate materials in a wide range of dental applications such as surgical defects, maintaining alveolar ridge height, furcation defects and as a bone void filler (Table 2) [153]. 

#### 3.2.7. Polymers

Synthetic polymers can be further classified into degradable and non-degradable subtypes. The most widely used polymers for bone regeneration include polylactic acid, polyglycolic acid, polyε-caprolactone and their copolymers and derivatives, collectively these are known as aliphatic polyesters [12,17]. The main advantages exhibited by this group of materials include their customisable forms, low immunogenicity, controllable resorbability, porosity, and physiochemical structure [12,154]. However, concerns relating to the release of acidic degradation products resulting in the alteration of local pH, osteoconductivity and poor cell adhesion capacity remain, thus restricting their use in the dental field (Table 2) [155,156]. Animal studies using polymer-based bone substitute materials have been conducted, and these have shown varying results, ranging from no complications in most cases to occasional inflammatory reactions [17]. It has been suggested that modifications to polymer-based scaffolds, such as the addition of HA or TCP may improve the bone regeneration potential of the resulting material [157,158]. A recent study found that loading VEGF using a silk fibroin coating onto the surface of a polymer-based bone substitute was able to achieve controlled release delivery of bioactive molecules, exhibited enhanced angiogenic properties and improved osseointegration into the graft site [159,160]. Another study also found that 3D printed polylactic acid-based biopolymer possessing 200 µm pore diameters exhibited improved cell proliferation and differentiation [161]. *HTR Synthetic Bone^TM^* is an example of a commercially available polymer-based bone substitute material composed of PMMA, polyhydroxylethylmethacrylate and calcium hydroxide. This product has been successfully used in the management of periodontal intrabony and furcation defects (Table 2) [29,162].

#### 3.2.8. Metals

**Figure 4:**
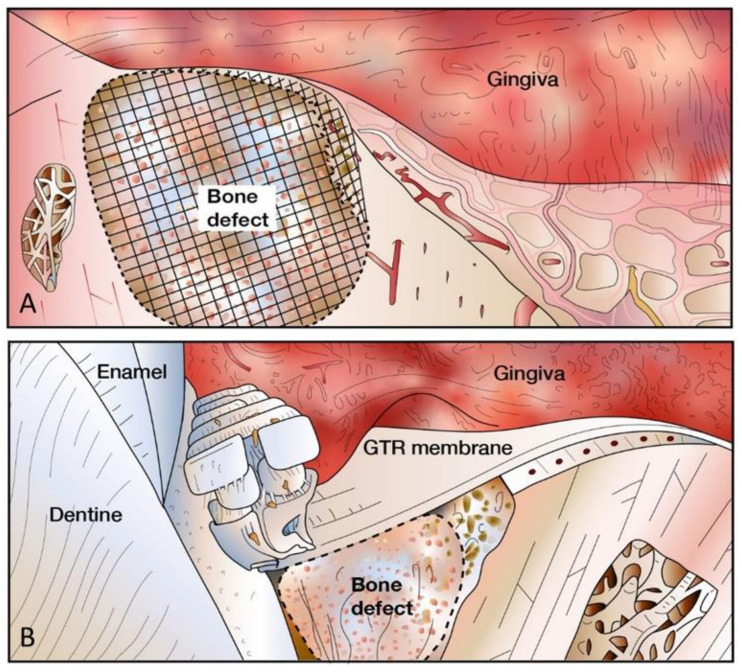
(**A**) Use of a titanium mesh as a structural scaffold and physical barrier in GBR for prevention of soft tissue cell migration and promotion of bone regeneration. (**B**) Use of a barrier membrane in GBR.

Recent research has identified a role of metallic ions, such as magnesium (Mg), strontium (Sr), zinc (Zn) and silicon (Si) in the maintenance of bone and stimulation of osteogenesis [11]. In the dental field, the use of nickel-titanium materials for bone regeneration has been explored due to their numerous desirable properties, including good mechanical strength, good biocompatibility, corrosion resistance and elastic modulus (Table 2) [163]. Studies have found that using a nickel-titanium membrane with pore sizes between 50 and 125 µm has resulted in vascularization and bone healing. The membrane serves the purpose of providing a physical barrier preventing the migration of epithelial cells and fibroblasts, selectively allowing the migration of osteogenic progenitor cells to the defect site for new bone formation (Figure 4) [164]. The primary role played by the nickel-titanium membrane is that of a structural scaffold, providing a support structure for cell adhesion, proliferation and differentiation, resulting in the formation of new bone. The major drawbacks of using nickel-titanium membranes include the need for a second surgical procedure and the possibility of soft tissue dehiscence and exposure of the membrane [164]. Over the years, titanium membranes have been used in bone reconstruction in alveolar bone defect sites; to stabilize the placement of autograft materials; as an adjunct for other grafting materials and as a membrane barrier for GBR (Table 2) [163].

In recent years, Liu et al. have developed a magnesium-based bone substitute fabricated using pure Mg (99.9%) and a Mg-30wt% Sr alloy in a high-purity graphite crucible generated in a mixed gas atmosphere. This material utilized the combined properties of degradability, excellent mechanical properties and biocompatibility of pure Mg and Mg-Sr alloys [165,166]. The authors reported that when compared with conventional commercial bone grafts, such as calcium sulfates, HA, and TCP materials, this new Mg-based material displayed improved tensile and compressive strengths, improved biocompatibility and improved antibacterial properties. These finding ins indicate its potential use in load-bearing areas as a bone substitute material [167]. 

### 3.3. Composite Bone Substitute Materials 

Composite bone substitute materials aim to improve the mechanical properties of the resulting combination of different materials, such as bioglass and polymers through combining their osteoconductive properties. They are commonly used to expand the utility of autograft products and are often combined with bone marrow or act as carriers for BMPs to improve their osteoconductive and osteoinductive properties [10,17]. Composite bone substitutes combining two or more materials have been used to exploit the advantages of various materials [168]. 

*NanoBone^TM^* is a relatively new composite bone substitute that combines 76% w/w nanocrystalline HA and 24% w/w silicon dioxide [17]. The silicon dioxide component mediates the remodeling of bone via the surface adhesion of autologous proteins. *NanoBone^TM^* has a highly porous structure yet maintains high fracture toughness and mechanical strength. It has a rapid mode of action and can rapidly integrate into the host tissue. Studies have observed new trabecular bone formation in animal models, followed by resorption of the composite material following complete bone regeneration after eight months [110,169]. Further studies in human subjects have found that *NanoBone^TM^* can preserve alveolar bone height at extraction sites. When used with platelet-rich fibrin, it can accelerate bone regeneration and improve the quality and quantity of newly formed bone following excision of mandibular cysts (Table 2) [170,171].

Another commonly used resorbable composite bone substitute product in dentistry is *Fortoss Vital^TM^* which is a biphasic alloplastic material composed of β-TCP within a calcium sulphate matrix [17,172]. When the two components are mixed, this produces a workable paste that hardens in a self-setting manner allowing for high adaptability to defect sites. This material acts as an osteoconductive structural scaffold, possessing a negative surface charge that is able to attract positively charged host BMPs and interstitial fluid, which then in turn leads to the recruitment of osteoblasts to the graft site ultimately resulting in improved bone regeneration [172]. Additionally, upon the setting of the material, a membrane forms acting as a barrier and preventing the infiltration of unwanted cells, selectively allowing the migration of osteogenic cells to mediate bone regeneration (Figure 4) [173,174,175]. In dental applications, *Fortoss Vital^TM^* has been successfully used in a variety of procedures such as alveolar bone augmentation, implant rehabilitation and socket preservation which have shown profound bone regeneration following grafting with *Fortoss Vital^TM^* (Table 2) [112,172]. In general, composite bone substitutes have performed well clinically and are regarded as promising alternatives to autograft materials [17]. 

### 3.4. Growth Factor-Based Bone Substitutes (GFBSs)

Growth factors (GFs) such as BMPs, platelet-derived growth factors (PDGFs) and insulin-like growth factors (IGFs) have been found to possess osteoinductive properties, allowing for accelerated bone regeneration in bony defects [176]. In the dental field, the first use of bioactivated materials with growth factors is in the use of plasma rich in growth factors (PRGF), platelet rich plasma (PRP) and plasma rich in fibrin (PRF) to accelerate bone healing in patients with bisphosphonate related osteonecrosis of the jaw (BRONJ) [177,178]. Recent studies have revealed varying results which cast doubt on whether the additional use of PRP with other grafting materials in the treatment of infrabony defects and sinus augmentation provides any added benefits [89,177,179,180,181]. Since the early 2000s, BMP-2 and BMP-7 have been the most commonly used USFDA approved growth factors for bone grafting procedures in dentistry and function as the active components of two major commercial products—*Infuse*^TM^ and *Osigraft^TM^*, respectively [182]. However, the production of *Osigraft*^TM^ has since been halted and the use of *Infuse*^TM^ has been associated with a large number of life-threatening complications [182]. Although the use of GFBSs has presented a promising area for the introduction of new bone substitutes, novel bioactivated products with GFs have generally not progressed beyond the animal study stage in recent years. The products which have progressed beyond this stage, such as *Augment*^TM^ have used recombinant growth factors derived from human platelets (rhPDGF-BB) and rhBMPs [178]. These products are generally used in combination with a structural scaffold or carriers such as allograft material, collagen sponges, titanium mesh or β-TCP/HA [178,182]. The use of bioactivated products with growth factors have been indicated for use in bilateral maxillary sinus augmentation, ridge augmentation, coating on implants and bony and ridge defects [178]. Currently, several concerns remain regarding the effective use of GFBSs in targeted bone regeneration. These include the need for the inclusion of a structural scaffold due to the lack of osteoconductive capability of GFBSs and the need for GFs to reach the target tissue whilst retaining their bioactivity during the therapeutic time frame [176]. The development of a delivery method that is able to satisfy both these needs simultaneously has been regarded as extremely challenging to the point of being near-impossible [182]. Strategies which address these concerns that have been proposed include entrapment of GFs within the scaffold, covalent or non-covalent binding of GFs to the scaffold and the use of micro/nanoparticles as GF reservoirs allowing for prolonged controlled release over time [176]. 

Sticky bone is another recently developed concept which utilizes a bone graft matrix enriched with growth factors using autologous fibrin glue [183,184]. The use of sticky bone is able to stabilize bone graft material in bony defects allowing for accelerated bone regeneration and minimizing bone loss. The advantages of this material include good moldability, good structural stability, selectivity for osteogenic progenitor through prevention of soft tissue cell migration via fibrin interconnections; and fibrin network allowing for rapid cell adhesion and accelerated healing [183]. When used in combination with a concentrated growth factor (CGF) membrane, or titanium mesh, grafting with sticky bone in an atrophic alveolar ridge resulted in favorable three-dimensional ridge augmentation over a 4-month period [183]. 

### 3.5. Bone Substitutes with Infused Living Osteogenic Cells 

Viable osteogenic progenitor cells, such as MSCs, can be used alone or in combination with other materials such as cytokines, GFs and scaffolding carriers and carriers including DBM to stimulate new bone formation and enhance bone healing through osteoconduction and osteogenesis. MSCs are non-hematopoietic multipotent cells routinely derived from bone marrow [185]. They are able to differentiate into osteogenic cells and can regenerate large bone defects when used with a scaffolding carrier [186,187]. Studies have shown that bone substitute materials that are bioengineered with MSCs can markedly improve bone healing and reconstruction when compared with the use of MSCs alone or a bone substitute material in the absence of MSCs. The resulting new bone formed displays significantly improved biomechanical performance and thus improves the rates of successful placement of dental implants [188]. Direct infusion with MSCs can promote more rapid and consistent bone healing [189]. 

In the dental field, various preclinical studies investigating the use of multipotent stem cells for periodontal regeneration have been conducted. Cao et al. and Hu et al. demonstrated that the use of heterologous MSCs derived from the dental pulp of extracted third molars in periodontal defects, whether in the form of cell sheets or cell injections were able to significantly increase regeneration of alveolar bone heights by 52.7 mm and 32.4 mm, respectively, in experimental pig models [190,191,192]. The difference in the increase in bone heights observed is potentially due to the ability of the 3D structure of cell sheets to mimic the physiological function of structural scaffolds [192]. Additionally, Park et al. found that the use of MSCs derived from heterologous periodontal ligament tissue and applied to periodontal defects increased alveolar bone regeneration to a greater degree than MSCs derived from heterologous dental pulp in experimental dog models [192,193]. Clinically approved products available commercially for dental use include *Bioseed-Oral Bone^TM^* and *Osteotransplant DENT^TM^*, which utilize an autologous source of MSCs with an appropriate scaffold [194]. These products are indicated for use in sinus augmentation of severely atrophic maxilla to achieve predictable implant placement [195]. 

Despite the many benefits presented by products infused with stem cells, various limitations persist such as low survival rates of stem cells immediately following transplantation, high self-cost and complexity of procedures, manufacturing difficulties related to autogenous cells, need for special storage conditions such as below −80 °C, long wait times and processing periods and legal regulation. Due to these difficulties, the use of stem cell-infused bone substitutes is currently not routinely applied and is restricted to the use for specific indications [194]. 

## 4. Future of Bone Substitute Materials in Dentistry

Despite the establishment of criteria defining the ideal bone grafting material several decades ago, to this day, autografts remain the gold standard and only material that possesses all four fundamental biological properties [68]. However, their limited availability and other associated limitations discussed previously have driven a shift towards using alternative grafting materials and the development of novel synthetic bone substitutes. Although there have been tremendous efforts to meet this need, all currently available materials on the market still demonstrate a shortcoming in biomechanical performance [17].

Producing a mechanically strong, interconnected porous structure allowing for ideal osseointegration and vascularization has been identified as the major difficulty faced in material development. Regrettably, synthetic bone substitutes only possess osteoconductive properties where bone regeneration is restricted to the outer surface layer [68]. This supports the need for the careful structural design of new materials including considering vital biological parameters such as pore size, density, morphology and interconnectivity and resorbability [12]. There has been a recent trend for the incorporation of osteoinductive growth factors and/or MSCs with a structural scaffold to increase the material’s bone regenerative potential and inhibit undesirable inflammatory recipient responses. Additionally, there has also been increasing interest in the controlled time-release delivery of growth factors as a means of maintaining their bioactivity over the therapeutic window [176]. Thus, the development of novel grafting materials should focus on incorporating as many ideal biological parameters as possible, whilst ensuring that such materials would be readily available, cost-effective and clinically evidence based. 

Another major challenge we face is the lack of research investigating the safety and efficacy of newer bone grafting materials [182]. Most of the information available regarding these newer materials are derived from case reports or experimental animal models, and thus the reliability of this information may be questionable. More standardized preclinical and clinical studies will need to be performed and documented better to understand each material’s clinical viability and benefits to introduce more commercially available products. To better understand the clinical viability and benefits of each material to introducing more commercially available products. 

## 5. Conclusions

Bone graft and substitute materials which are either in the form of particulate or blocks are mostly used in dentistry to regenerate the missing hard tissue structures. There is a high and growing demand for new and more efficient dental grafting materials. Current bone graft and substitute materials primarily serve as a structural framework for osteo-regenerative processes that only satisfy the osteoconductivity criteria. Additionally, potential issues persist relating to graft vs. host responses for all current non-autograft-derived materials. However, as the research in the field of tissue engineering progresses there have been many new developments such as diverse ceramic and polymeric-based bone substitutes integrated with growth factors or modified with living osteogenic progenitor cells. Our understanding of these materials and the growth factors at the molecular level is growing, which allows us to better control and modify their structure, understand their surface properties, and tune the interaction with other materials or physiological environment. This progress will eventually allow us to design and develop more effective dental bone substitutes. Nevertheless, the cost of these bone substitutes is another aspect. Clinicians should consider the higher costs of these new technologies in comparison with the benefit of existing osteoconductive only implants. Due to continual technological advancements in this field, natural bone grafts have gradually been replaced by synthetic bone substitutes. Development of hybrid grafts which utilize growth factors and living osteogenic cells capable of inducing bone regeneration presents the future of dental bone grafting and dental implants. Good examples include bone substitutes that can release bone morphogenic proteins or platelet-derived growth factors in a controlled manner. Despite the progress highlighted in this review article more work is needed to develop dental biomaterials that have a porous structure, mechanically stability, controlled degradation, and remodeling ability which is comparable with the rate of new bone formation.

## Figures and Tables

**Table 1:** Characteristics of commercially available natural bone graft and substitute materials.

Material Type	Product Name	Material Source	Forms Available	Clinical Applications	Advantages	Limitations	Type of Study and Outcome	Reference
**Cortical Allograft**	*MinerOss Cortical^TM^*	Mineralized cortical allograft	Fresh, frozen, freeze-dried Whole bone segments, block, pieces	Alveolar ridge augmentation Periodontal osseous defects Sinus augmentation	Osteoconduction Osseointegration Avoids donor site morbidity	Risk of disease transmission Immunogenicity	*Clinical trial* Bone formation 6 months following sinus augmentation procedures. Average of 3.5 mm horizontal ridge width gain, 4 months following placement of FDBA	[21,22]
**Cancellous Allograft**	*MinerOss Cancellous^TM^*	Mineralized cancellous allograft	Fresh, frozen, freeze-dried Chips, wedges, pegs, powder	Cleft repair	Osteoconduction Osteoinduction Osseointegration Avoids donor site morbidity	Same as cortical allograft		
**Demineralised Bone Matrix**	*Dynagraft D Putty^TM^* *Opteform^TM^* *Grafton DBM^TM^*	Human DBM	Putty, moldable pastes, blocks, particulates, powder	Bony void filler Periodontal osseous defects Sinus augmentation	Osteoinduction Osteoconduction Ease of handling Low immunogenicity Avoids donor site morbidity	Poor mechanical strengths Osteoinductive potential can be affected by tissue processing and host responses	*Clinical trial* 50–60% resolution of periodontal intrabony defects Remineralization and new bone formation following sinus augmentation with DBM	[23,24]
**Deproteinised bovine bone**	*BioOss^TM^* *OsteoGraf^TM^* *Cerabone^TM^*	Bovine	Block, granules, particulates	Sinus augmentation Socket/ridge preservation Horizontal and vertical augmentation Peri-implant defects	Good osteoconduction Very similar structures and biomechanical properties to human bone Low immunogenicity	Brittle Lacks fracture toughness	*Clinical Trial* New bone formation, intermingled with *BioOss^TM^* particles 6-7 months following graft placement 14/14 implants placed in patients with insufficient alveolar ridge width in the maxillary lateral incisor region successfully osseo integrated and were functionally stable	[25,26]
**Algae-based**	*Algipore^TM^*	Red algae	Granules	Alveolar bony defect filler Preservation of ridge height	Osteoconduction Good resorbability Large surface area for protein adhesion Low immunogenicity Resorbability	Lack of studies investigating use in humans	*Clinical trial* 95% implant survival rate in atrophic maxilla grafted with Algipore 14 years following graft placement New bone formation around and within the pores of implanted *Algipore^TM^* particles, 7 months following graft placement	[27,28]
**Coral-based**	*ProOsteon^TM^* *BioCoral^TM^* *InterPore^TM^*	Marine coral	Block, Granules	Sinus augmentation Periodontal osseous defects Restoration of alveolar ridges	Osteoconduction Good compressive strength Improved cell adhesion Low immunogenicity	Brittleness Poor resorption Low tensile strength	*Clinical trial* Decrease in periodontal probing depths and gingival recession 5 years following grafting with BioCoral Bone formation within, and along the walls of the pores of grafted *Interpore 200^TM^,* starting 3 months and continuing beyond 6 months following graft placement in periodontal osseous defects of three recipients	[29,30]

FDBA: freeze-dried bone allograft; DBM: demineralized bone matrix.

**Table 2:** Characteristics of synthetic bone grafting materials.

Material Type	Product Name	Forms Available	Indications	Advantages	Limitations	Type of Study and Outcome	Reference
Hydroxyapatite	*Ostim^TM^* *Endobon^TM^*	Blocks, wedges and granules	Intraosseous defects Furcation defects Socket preservation Horizontal or vertical augmentation in non-stress bearing areas Periodontal osseous defects	Osteoconduction Macroporous structure comparable to human bone Biocompatibility Excellent hydrophilicity for vessel uptake	Donor site morbidity Low mechanical strengths Delayed resorption rate Limited availability	*Clinical trial* Significant bone regeneration in 2 and 3-wall intrabony periodontal defects 6 months following placement of *Ostim^TM^* graft Decreased periodontal pocket depth, decreased clinical attachment loss, decreased intrabony defect depth, 6 months following placement of *Ostim^TM^* graft	[93,94]
Tricalcium phosphate ceramics	*Cerasorb^TM^* *OSferion^TM^* *Orthograft^TM^*	Blocks, cylinders, wedges, granules	Void filler for alveolar, periodontal, periapical, peri-implant and cystic defects	Osteoconduction Ease of handling Radiopacity allowing monitoring of healing Good resorbability Low immunogenicity	Poor mechanical properties in particular compressive strength	*In vivo (goat)* Bone regeneration comparable to that of autografts in alveolar clefts, 6 months following placement of β-TCP *Clinical trial* Successful osseointegration and prominent bone formation along graft surface evident 28 days after placement of *OSferion^TM^*	[95,96]
Biphasic calcium phosphate ceramics	*MASTERGRAFT^TM^*	Moldable putty, granules	Void filler for alveolar, periodontal and cystic defects Preservation of sockets Ridge augmentation Maxillary sinus lifting Periapical surgery	Osteoconduction Osteoinduction Resorbability Comparatively greater mechanical strengths than either TCP or HA alone	Compressive strength remains lower than that of cortical bone	*Clinical trial* New bone formation with histological observation of osteogenic activity surrounding *MASTERGRAFT* granules, 4-5 months following graft placement New bone formation and minimal ridge width reduction observed in post-extraction alveolar ridges of 15 patients	[97,98]
Bioglasses	*Perioglas^TM^* *Biogran^TM^*	Particulates	Periodontal defects Furcation defects Socket preservation Cystic defects Fenestration and dehiscence defects	Osteoconduction Biocompatibility Antimicrobial activity Porous structure Completely resorbable	Brittle Low mechanical strength Poor fracture resistance	*Clinical trial* 88.6% success rates of implants placed in sites grafted with bioactive glasses, 29 months following bioglass material Decreases in periodontal pocketing depth, clinical attachment loss, gingival recession, depth of bony defect observed, 9 months after placement of *Perioglas^TM^* either alone, or in combination with a non-resorbable membrane *GoreTex^TM^* or bioresorbable membrane *Resolut Adapt^TM^*	[99,100,101]
Calcium phosphate cements	*Norian^TM^* *ChronOS inject^TM^* *Hydroset^TM^* *BoneSource^TM^*	Injectable paste, moldable putty	Bony defect filler Reconstruction of bony fractures Implantology	Osteoconduction Self-setting ability Mouldability Biocompatibility	Low speed of cell adhesion Brittle Concerns relating to extrusion of material to adjacent tissues	*Clinical Trial* Nearly complete bone regeneration in alveolar ridge defects, 6 months following placement of CPC material *Case Report* Complete replacement by newly formed bone of *Norian^TM^* graft placed in a large 3-wall mandibular defect, one year following graft placement	[102,103]
Calcium sulfates	*OsteoSet^TM^*	Various sizes pellets	Void filler for surgical defects and furcation defects Preservation of sockets and alveolar bone heights	Osteoconduction Low cost Readily available High mouldability Biocompatibility Short setting time	Rapid resorption which is faster than that of human bone Relatively high risk of infection and inflammation	*Clinical trial* When used in combination with FDBA, resulted in the reduction of periodontal probing depths, gains in clinical attachment, defect fill and resolution, 12 months following placement of calcium sulfate graft material *Double-blind randomized trial* 42% of bony defect filled with new bone, 6 weeks after placement of *OsteoSet^TM^* graft. No statistically significant additional bone formation observed during 3-6 months period.	[104,105]
Polymers	*Bioplant HTR Synthetic Bone^TM^*	Particulates, granules, ready to use in syringe	Ridge augmentation and preservation Furcation defects	Osteoconductive Biocompatible Customizable forms Low immunogenicity Porous structure Radiopaque	Concerns relating to acidic degradation products	*Clinical trial* Reduction in periodontal probing depths, clinical attachment gain and significant resolution of defects in alveolar crest bone, 6 months following placement of *Bioplant HTR Synthetic Bone^TM^* Decreased periodontal probing depths, mean horizontal and vertical furcation probing attachment levels, six years after placement of *Bioplant HTR Synthetic Bone^TM^*	[106,107]
Metals	*OSS Builder^TM^*	Mesh/membrane available in lateral and papilla design forms	Lateral forms—horizontal or vertical bone augmentation Papilla forms—restoring papilla height for aesthetics	Osteoconduction, acts as a membrane barrier for GBR Good mechanical strength Good biocompatibility Corrosion resistance Porous structure enhancing cell adhesion	Need for a second surgical visit Possibility of soft tissue dehiscence and exposure of the membrane	*Clinical trial* Significant bone formation in alveolar ridge, 4 months following placement of autograft with titanium mesh *Case Report* Increase in alveolar crestal bone width and height observed, 5 months after placement of autograft mixed with equine-derived xenograft and a titanium mesh	[108,109]
Composites	*NanoBone^TM^* (nanocrystalline HA/silicon dioxide)	Putty, granulate, block, ready to use “QD”	Bone void filler Socket preservation	Osteoconduction Osteoinduction Resorbability Moldability Good cell adhesion	Lack of studies investigating use of *NanoBone^TM^* in humans	*In vivo (mouse)* New trabecular bone formation, followed by resorption of graft material, 8 months following placement of *NanoBone^TM^In vivo (dog)* Significantly greater amount of new bone formed in extraction sockets observed at 45 and 90 days after placement of *NanoBone*^TM^ with PRF than *NanoBone^TM^* alone or in the control group	[110,111]
	*Fortoss Vital^TM^* (β-TCP/calcium sulphate)	Paste	Alveolar bone augmentation Implant rehabilitation Socket preservation	Osteoconduction Osteoinduction Fully resorbable Moldability Porous structure Good cell adhesion	Contact with blood will delay setting time of the paste	*Clinical trial* Formation of new viable bone, 12 weeks after placement of *Fortoss Vital^TM^* Reduction in periodontal pocketing depth, clinical attachment loss, but increases in gingival recession observed 2 years after placement of *Fortoss Vital^TM^*	[112,113]
	*SmartBone^TM^* (DBM/polymer/collagen)	Blocks, microchips, plate, granules, wedge, cylinder, rod	Periodontal osseous defects Socket preservation Alveolar ridge augmentation Sinus augmentation	Similar morphology to human bone Rapid blood cell adhesion and proliferation due to high hydrophilicity Improved volumetric stability High load resistance for large bony defects	Comes in single use only packages	*Clinical trial* Formation of new bone, and increases in alveolar bone dimension, 4 months following placement of *SmartBone^TM^* Successful osseointegration and new bone formation observed surrounded by vascular connective tissue, 4 months following placement of *SmartBone^TM^* graft.	[114,115]

FDBA: freeze-dried bone allograft; GBR: guided bone regeneration; TCP: tricalcium phosphate; PRF: platelet-rich fibrin; DBM: demineralized bone matrix; HA: hydroxyapatite; CPC: calcium phosphate cements.

## Data Availability

The data presented in this study are available on request from the corresponding author.

## Sample Availability

Not available.
